# Stress stability study of simeprevir, a hepatitis C virus inhibitor, using feasible TLC-spectro-densitometry: application to pharmaceutical dosage form and human plasma

**DOI:** 10.1039/d0ra01172j

**Published:** 2020-06-03

**Authors:** Bassam Shaaban Mohammed, Amal E. Hamad, Sayed M. Derayea

**Affiliations:** Department of Pharmaceutical Analytical Chemistry, Faculty of Pharmacy, Menoufia University Shebin El-Kom Menoufia Egypt Bassam.shaaban@phrm.menofia.edu.eg; Department of Analytical Chemistry, Faculty of Pharmacy, Minia University Minia 61519 Egypt

## Abstract

Simeprevir is one of the newest direct action anti-hepatitis C drugs. In the present work, a simple, highly selective and stability-indicating, high-performance thin-layer chromatography (HPTLC) method is proposed and validated for the assay of simeprevir both in pharmaceutical dosage form and spiked human plasma. The method used silica gel 60 F_254_ coated HPTLC aluminum plates as the stationary phase. The mobile phase system was ethyl acetate-hexane-methanol (5 : 4 : 1, v/v/v). The wavelength for both detection and quantitation was set at 288 nm. This system was found to give a compact spot of simeprevir; the retardation factor (*R*_F_) value was 0.67 ± 0.02. The guidelines of the International Conference on Harmonization were followed to validate the proposed analytical method, and the results were acceptable. The calibration curve was linear over the range of 80–1000 ng per spot. The limit of detection was 19.0 ng per spot, and the limit of quantitation was 57.0 ng per spot. The drug was subjected to various stress conditions including hydrolytic, oxidative and UV-induced resulting in varying degrees of degradation. The results showed that the proposed method could efficiently separate the degradation products from the intact drug and allow its satisfactory quantitation. The proposed method was employed successfully for the accurate and reproducible analysis of the pharmaceutical preparation and human plasma containing the drug. The proposed method's precision and accuracy were statistically similar to those of a reported method.

## Abstract 2

Simeprevir is one of the newest direct action anti-hepatitis C drugs.

## Graphical abstract

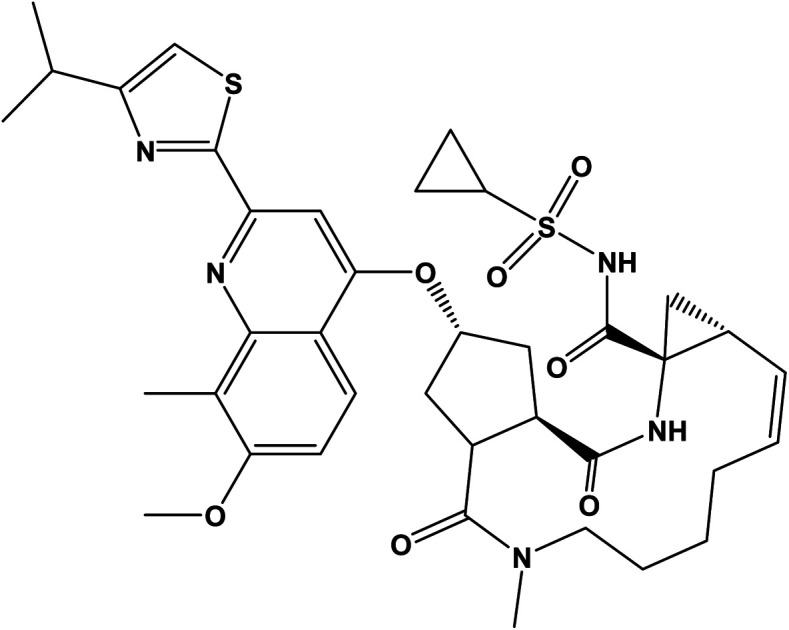

## 1. Introduction

**Fig. 1:**
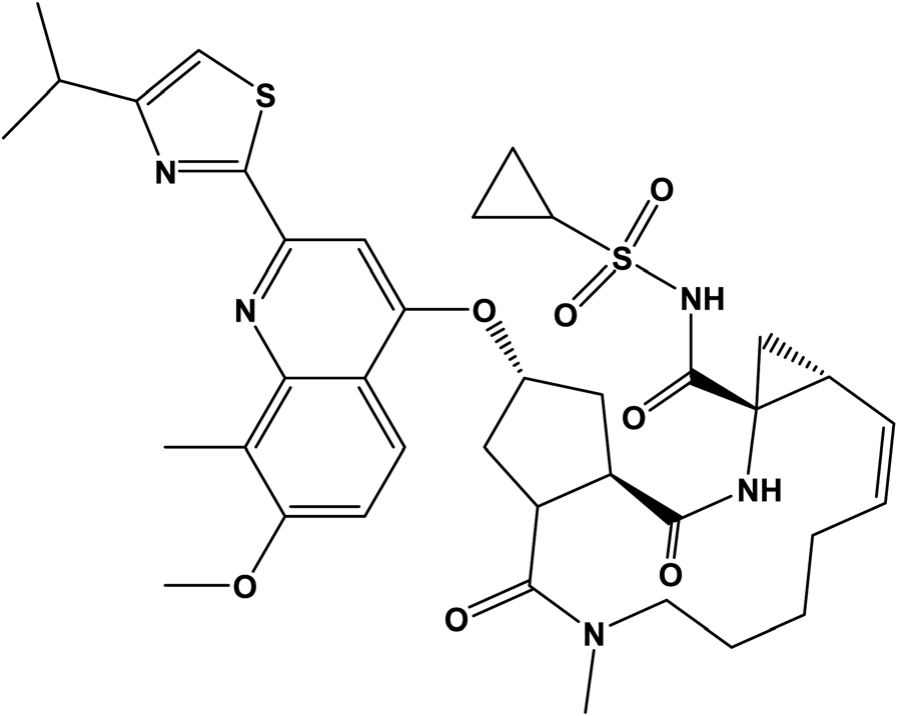
The chemical structure of simeprevir (SMV).

More than one hundred and fifty million people worldwide have been affected by HCV infection which represents the principal cause for hepatocellular carcinoma and liver failure.^1^ Many years ago, the combination of ribavirin and pegylated interferon *α* was the standard treatment for achieving a sustained virological response in ≥80% of patients with HCV genotypes 2 and 3 but only ∼50% in genotype 1 patients. However, it caused significant side effects.^2,3^ Great progress was brought about with the emergence of direct-acting antivirals which attack the viral nonstructural proteins. SMV inhibits N3S/4A protease enzyme and is orally administered once-daily for the treatment of chronic HCV genotype 1 infection in combination with other antiviral agents showing a highly sustained virological response rates in patients with HCV genotype 1 infection during phase II and III trials.^4^ SMV (Fig. 1) is a white powder soluble in dichloromethane, slightly soluble in acetone, very slightly soluble in ethanol; practically insoluble in water and propylene glycol.^5^

Due to its recent market entry, not many analytical methods were found in the literature. The reported methods of analysis included; four liquid chromatographic methods for estimation of simeprevir in plasma samples using tandem mass detection,^6,7^ UV detection^8^ and diode array detection.^9^ Other non-chromatographic methods were also reported; synchronous spectrofluorimetric^10^ and spectrophotometric methods.^11,12^ HPTLC as a chromatographic technique shares the advantage of being able to separate and analyses complex mixtures. Although it's not as sophisticated or sensitive as HPLC, yet it possesses several strengths over HPLC such as simplicity and speed as well as great reduction in the analysis cost and time as it does not require expensive equipment nor tedious and time consuming sample pre-treatment steps. Furthermore, it uses much less mobile phase and sample. Therefore, the present work aimed to develop a new HPTLC-densitometric method for the determination of simeprevir in dosage forms and human plasma samples. The proposed method was developed also to study and evaluate degradation stability of SMV since it was able to accurately determine SMV in presence of its various degradation products without any prior extraction.

## 2. Experimental

### 2.1 Instrumentation

An HPTLC system with the following specifications was used: CAMAG Linomat V sample applicator (Muttenz, Switzerland) CAMAG 100 μL sample syringe (Muttenz, Switzerland); band width, 3 mm; application rate 15 s μL^−1^, slit dimension, 3 × 0.45 mm; and scanning speed, 20 mm s^−1^. Densitometric scanning was performed using a CAMAG TLC Scanner 3, operated by win CATS evaluation software (version 1.4.4.6337).

Super-mixer (GEMMY Industrial CORD, Taiwan) and Bath Sonicator (Sonicor SC-101TH) were also used. All weighing were performed on an electronic single pan balance (Precise XB 220A, 10 mg to 220 g Switzerland).

### 2.2 Chromatographic conditions

The sample was applied on HPTLC aluminum plates pre-coated with silica gel 60 F_254_. The mobile phase consisted of ethyl acetate-hexane-methanol (5 : 4 : 1, v/v/v). The samples were applied as bands of 3 mm long with 5 mm spacing under nitrogen stream. The chromatogram was developed in a linear manner to a distance of 9 cm in 20–20 cm twin-trough TLC chamber (CAMAG) at room temperature. The development chamber was first saturated for 30 min with the mobile phase prior to the development. The plates were then completely dried by an air dryer before being scanned with a CAMAG TLC Scanner 3 in the absorbance mode at 288 nm.

### 2.3 Materials and reagents

Aluminum plate precoated with silica gel 60 F_254_, (20 × 10 cm) with 250 mm thickness (Merck, Darmstadt, Germany). Pooled blank plasma was collected from healthy volunteers and kept in the refrigerator until needed. Pharmaceutical dosage form: Merospevir® capsules (batch number 160117) were kindly supplied by AUG PHARMA, Giza, Egypt. Each capsule was labeled to contain 150.0 mg of simeprevir.

All chemicals and reagents used throughout this work were analytical grade. SMV was kindly gifted by AUG PHARMA (6^th^ Industrial Zone – 6 October City – Egypt). Ethyl acetate, hexane, methanol, sodium hydroxide, hydrochloric acid, and hydrogen peroxide (30 volume) were purchased from El Nasr Chemical Co. (Abo-Zaabal, Cairo, Egypt).

### 2.4 Standard solution and calibration graphs

The SMV stock standard solution (100 μg mL^−1^) was prepared by dissolving an accurately weighed amount of SMV salt powder equivalent to 10.0 mg in a 100 mL volumetric flask using ethanol.

**Fig. 2:**
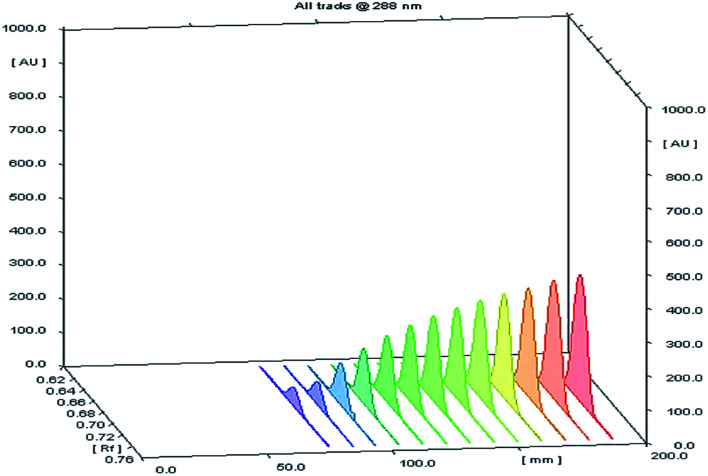
A 3D chromatogram for the calibration of SMV, using the optimized solvent system “ethyl acetate–hexane–methanol” in the ratios (5.0 : 4.0 : 1.0, v/v/v), and wavelength of TLC scanner was set at 288 nm.

Different volumes of the stock standard solution (0.8–10 μL) were spotted on the TLC plates that gave spot concentrations in the range of 80–1000 ng per spot (Fig. 2). The calibration graph was constructed by plotting area under peak against corresponding SMV concentration.

### 2.5 Preparation of plasma samples

The authors got the permission for using plasma sample of human volunteers from Minia Hospital according to the declaration of Helsinki, and International Conference on Harmonization (ICH) guidelines. All experiments were performed in compliance with the guidelines and approved by “The Commission on the Ethics of Scientific Research”, located in Faculty of Pharmacy, Minia University. An informed written consent was obtained from each participant. Drug-free human blood samples were obtained from healthy volunteers. 5 mL of which were centrifuged in a heparinized tube at 4000 rpm for 30 min. 1.0 mL aliquots of the supernatant were pipetted into 10.0 mL volumetric flasks and spiked with 1.0 mL of SMV solution containing 0.8–10 μg mL^−1^. Acetonitrile (2 mL) was used to precipitate protein. The mixture was diluted to the mark with ethanol giving a final SMV concentration of 80–1000 ng mL^−1^. The solution was centrifuged at 4000 rpm for about 20 min, and the clear colorless solution was subjected to HPTLC analysis. A blank was prepared by treating the drug-free blood sample in the same manner without adding any SMV.

### 2.6 Analysis of pharmaceutical dosage forms

The contents of ten capsules Merospevir® capsules were evacuated and their contents were accurately weighed and mixed well. An amount equivalent to 10.0 mg of SMV was accurately weighted and transferred into a 100 mL volumetric flask, where they were dissolved in about 50 mL of ethanol by sonication for 5 min, and completed to the mark with ethanol and mixed well. The flask contents were filtered, rejecting the first portion of the filtrate. Different volumes of the obtained filtrate were spotted on the TLC plates, giving a final concentration of SMV between 80–1000 ng per spot.

### 2.7 Procedures for forced degradation study

After degradation (procedures detailed in the following subsections), aliquots of the final solutions were applied to the HPTLC plate in triplicates of SMV concentrations within the linear range of the calibration and the chromatograms were developed as described under (Section 2.2). The degradation procedures were as follows.

#### 2.7.1 Hydrolytic degradation

An accurately weighed amount of 100 mg of SMV was dissolved in ethanol in a 100 mL volumetric flask 10.0 mL aliquots of this stock solution were forced to degrade under both acidic and alkaline conditions by refluxing with 2.0 mL ethanolic of 1.0 M HCl, or 1.0 M NaOH; respectively at 60 °C for 2 h in the dark. The reaction mixtures were neutralized and sonicated for 10 min before being quantitatively transferred into 100 mL volumetric flasks, completed to the mark with ethanol and mixed well giving a final SMV concentration of 100 μg mL^−1^. For neutral hydrolysis study, 10.0 mL aliquots of ethanolic 100 μg mL^−1^ SMV solutions were refluxed in the dark at 90 °C for 6 hours.

#### 2.7.2 Oxidative degradation

The oxidative degradation was performed by adding 2.0 mL of 30% v/v H_2_O_2_ to 10.0 mL aliquots of stock SMV standard solution in a round bottom flask. The mixture was refluxed at 60 °C for 2 h and then boiled to expel excess hydrogen peroxide. The resulting solutions were quantitatively transferred into 100 mL volumetric flasks where they were completed to the mark with ethanol and mixed well to get final SMV concentrations of 100 μg mL^−1^.

#### 2.7.3 UV-induced degradation

For UV degradation study, 10.0 mL aliquots of ethanolic 100 μg mL^−1^ SMV stock solution were transferred into 100 mL volumetric flasks and exposed to UV irradiation at 254 nm for 48 h in an UV chamber. The flasks were completed to the mark with ethanol and mixed well.

## 3. Results and discussion

The current study's aim was to develop and validate an efficient, fast and cost-effective method of analysis for the determination and stability testing of simeprevir with low impact on the environment. HPTLC densitometric technique delivered on the desired method objectives since it was able to separate multiple analyte simultaneously with little solvent consumption and very simple procedure for sample preparation. To attain good resolution with sharp symmetric beaks, and with acceptable *R*_F_ values, several chromatographic conditions had to be optimized;

### 3.1 Method optimization

#### 3.1.1 Mobile phase system

Several trials were executed to select the appropriate mobile phase comparing different developing systems of varying compositions. At first binary solvent systems, such as ethyl acetate–hexane (8 : 2, v/v) were examined, but the bands were not satisfactorily resolved. Ternary mobile phase systems were then tried including ethyl acetate–hexane–acetonitrile (8 : 1 : 1, v/v) and ethyl acetate–hexane–methanol (7.0 : 2.0 : 1.0, by volume) were investigated. Still, poor resolutions, band broadening and tailing were observed. By changing the ratio of ethyl acetate–hexane–methanol, the resolution was improved but with tailed bands to some extent until using the ratio of (5 : 4 : 1 v/v) which gave optimum resolution. Quantitative determination SMV was performed by scanning the bands at 288 nm, which gave *R*_F_ value of 0.67 ± 0.02.

#### 3.1.2 Scanning wave length

Since SMV has a strong absorption band with *λ*_max_ at 288 nm, this wavelength was used for the detection of SMV in this study to ensure maximum sensitivity and lowest LOD. Other wavelengths gave the lower sensitivity.

#### 3.1.3 Saturation time

To obtain more reproducible results with good *R*_F_ values, the identical degree of vapor saturation must always be reached during the development of the plate.^13^ Therefore, variable saturation times (10–30 min) were tested to select the most appropriate time for saturation. It was observed that good results were obtained upon waiting for saturation 15 min or more; therefore, 20 min was used to ensure proper saturation.

#### 3.1.4 Migration distance and development time

The solvent de-mixing might cause a solvent gradient up the plate, which may have an effect on separation and alter *R*_F_ values. Therefore, the distance of mobile-phase migration need to be standardized.^14^ The optimum migration distance for the proposed method was found to be 80 mm which gave the best resolution. This migration distance required approximately 15 min.

After optimization, the selected chromatographic conditions were applied for developing the plate spotted with the drug and scanning at 288 nm. It was found that the band of the drug was compact and symmetric with a *R*_F_ value of 0.67 ± 0.02.

In addition, using the same chromatographic conditions proved successful in the separation of degraded SMV samples components with high resolution and sharp, compact, and symmetric peaks for the intact drug as well as all its degradation products. Consequently, it was not necessary to change the mobile phase or other parameters for the purpose of studying the forced stability of the drug.

### 3.2 Validation of the proposed analytical method

The planned analytical technique was validated according to the International Conference on Harmonization (ICH) guidelines regarding linear range, limit of detection (LOD), limit of quantification (LOQ), accuracy, precision, robustness, and selectivity.^15^

#### 3.2.1 Linearity and range

Calibration graph was constructed by plotting the obtained area under peak (AU) *versus* corresponding SMV concentration within a specific range. A good correlation coefficient (0.9997) was observed over the concentration range 80–1000 ng per spot indicating excellent linearity of the proposed method. A summary for the analytical parameters of the proposed method is presented in (Table 1).

**Table 1:** Analytical parameters for the analysis of SMV by the proposed TLC method

Parameters	Value
Concentration range (ng per spot)	80–1000
Correlation coefficient (*r*)	0.9997
Determination coefficient (*r*^2^)	0.9994
Slope (*b*)	7.9134
Standard deviation of slope	0.076
Intercept (*a*)	1477.8
Standard deviation of intercept	45.103
Limit of detection (LOD), ng per spot	19.0
Limit of quantitation (LOQ), ng per spot	57.0

#### 3.2.2 Accuracy

**Fig. 3:**
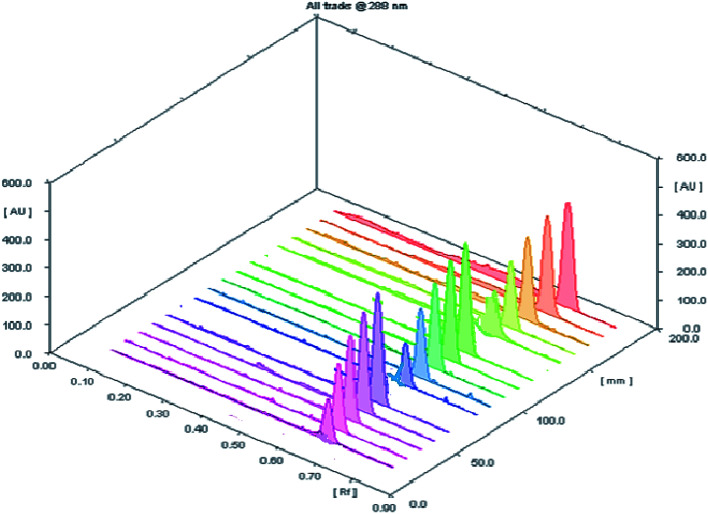
A 3D chromatogram for evaluation of the accuracy of investigated analytical procedure for determination of SMV at five concentration levels with three replicate measurements.

The accuracy of the planned HPTLC methodology was evaluated by direct analysis to five concentrations (100, 200, 400, 600 and 800 ng per spot) within the specified concentration range of the studied drug (three replicates for each concentration) (Fig. 3). The results of measurements were expressed as percent recovery ± standard deviation (Table 2). The obtained results have a close agreement between the measured and true values revealing a sufficient accuracy of the proposed procedure.

**Table 2:** Evaluation of accuracy of the investigated analytical procedure for determination of SMV at five concentration levels within the specified range

Sample number	Taken conc. (ng per spot)	Found conc.^a^ (ng per spot)	% recovery ± SD
1	100	100.63	100.64 ± 2.91
2	200	206.22	103.11 ± 0.36
3	400	389.51	97.37 ± 0.18
4	600	584.51	97.41 ± 1.51
5	800	790.20	98.77 ± 0.11

a Mean of three replicate measurements, (SD) standard deviation and (RSD) relative standard deviation.

#### 3.2.3 Precision

Precision was assessed through replicate analysis of SMV standard sample solutions at three concentration level (200, 400 and 600 ng per spot) on successive three times in the same day for intra-day precision (repeatability) and over a period of three successive days for inter-day precision (intermediate). The results of both levels of precision were presented in (Table 3). The calculated relative standard deviations of different measurements were all below 3%, indicating good precision at both levels of repeatability and intermediate precision.

**Table 3:** Evaluation of the intra-day and inter-day precision of the proposed TLC analytical method for determination of SMV in pure form

Precision level	Conc. (ng per spot)	% recovery^a^ ± RSD
Intra-day precision	200	100.80 ± 1.65
	400	100.74 ± 2.84
	600	99.59 ± 1.80
Inter-day precision	200	97.99 ± 1.78
	400	99.65 ± 1.96
	600	99.52 ± 2.23

a Mean value of three determinations.

#### 3.2.4 Detection and quantification limits

In order to estimate the sensitivity of the developed procedure, the limit of detection (LOD) and limit of quantitation (LOQ) were calculated. The following formulas were used; LOD = 3.3*σ*/*S* and LOQ = 10*σ*/*S* where (*σ*) is the standard deviation of the response of the blank and (*S*) is the slope of the calibration curve. The LOD and LOQ for SMV were 19.0 and 57.0 ng per spot, respectively (Table 1).

#### 3.2.5 Robustness

Robustness of the projected methodology was studied by little however deliberate variations within the optimized method parameters while running the proposed method on SMV concentration of 200 ng per spot. Variation in composition of the mobile phase (±0.1 mL), duration for chamber saturation (±5 min), time from spotting to development (10 min, 20 min and 30 min) and time from development to scanning (5 min, 15 min and 30 min) were all carefully studied. The effects of these changes on *R*_F_ values and peak area were evaluated by calculating the relative standard deviation (RSD) for each parameter in comparison with the optimally used conditions. The calculated standard deviations of different measurements were all below 3%. It was observed that, by slight variation of the proposed method variables, the (RSD) was in the range from 0.27–2.39. Pointing out that the good robustness and reliability of the proposed method for analysis of the drug (Table 4).

**Table 4:** Robustness testing for the TLC method of SMV (200 ng per spot)

Parameters	Factor	% recovery ± RSD
Mobile phase composition	5.0 : 4.0 : 1.0 (v/v/v)	Optimized
	5.1 : 4.1 : 1.1 (v/v/v)	99.92 ± 1.54
	4.9 : 3.9 : 0.9 (v/v/v)	100.05 ± 1.06
Saturation time	20 min	Optimized
	25 min	99.88 ± 2.39
	15 min	100.01 ± 0.27
Standing time after spotting	20 min	Optimized
	10 min	100.09 ± 1.90
	30 min	100.10 ± 2.19
Time from development to scanning	15 min	Optimized
	10 min	99.91 ± 1.82
	30 min	99.97 ± 0.56

### 3.3 Analysis of the pharmaceutical sample

**Fig. 4:**
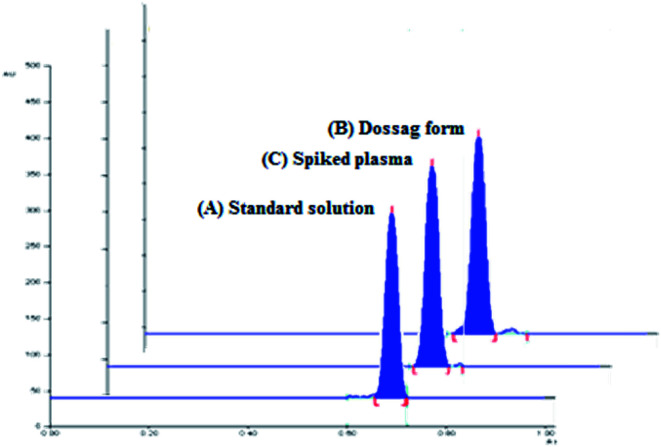
Two-dimensional TLC-densitograms of simeprevir; (A) standard solution (500 ng per spot), (B) extract of pharmaceutical dosage form (500 ng per spot), (C) plasma sample spiked with (500 ng per spot), using solvent system containing “ethyl acetate–hexane–methanol” in the ratios (5.0 : 4.0 : 1.0, v/v/v), and wavelength of TLC scanner was set at 288 nm.

The densitometric responses (peak area) from the standard and dosage form sample solutions were used to quantify the amounts of SMV in Merospevir® capsules. The results of the proposed technique were then statistically compared to that of the reference method.^10^ No significant difference was found at 95% confidence level (Table 5), this clearly showed that the proposed method could be reliably used for assay of SMV in its dosage form without interference from excipients two-dimensional TLC-densitograms of SMV in dosage form are presented in (Fig. 4B).

**Table 5:** Determination of the studied drug in Merospevir®150 mg per capsule form using the proposed and reported methods

Sample number	Taken conc. (ng per spot)	% recovery^a^	
		Proposed method	Reference method [10]
1	300.00	100.15	98.43
2	400.00	99.88	97.89
3	500.00	102.18	101.34
4	600.00	102.59	102.49
5	700.00	98.82	98.55
Mean ± SD		100.72 ± 1.60	99.76 ± 1.82
*F*-Value^b^			0.77
*t*-Value^b^			0.94

a Average of five separate determinations ± standard deviation.

b Theoretical values at 95% confidence limit: *t* = 2.31, *F* = 6.39.

### 3.4 Application to biological fluid (spiked human plasma)

The pharmacokinetic parameters of SMV explaining the maximum plasma concentration (*C*_max_) of SMV after multiple 150 mg once daily oral doses, *C*_max_ was approximately 2.588 μg mL^−1^ at *t*_max_ (4–9 h).^4^ The high sensitivity of the proposed technique permitted the estimation of SMV in human plasma spiked with different concentrations of SMV within the specified range. According to (ICH) guidelines of bioanalytical method,^16^ the recovery percentages ranged from 88.44% to 101.69%. The results showed in (Table 6) explain the suitability of the proposed method for the analysis of human plasma containing SMV without any significant interference from plasma components. 2D TLC densitograms are presented in (Fig. 4C).

**Table 6:** Application TLC determination of SMV in spiked human plasma

Precision level	Conc. (ng per spot)	% recovery^a^ ± RSD
Intra-day precision	300	88.44 ± 2.46
	400	90.23 ± 0.98
	500	90.83 ± 4.00
	600	94.32 ± 0.62
	700	99.41 ± 4.68
Inter-day precision	300	89.75 ± 1.32
	400	89.70 ± 0.52
	500	92.67 ± 3.43
	600	94.67 ± 0.20
	700	101.69 ± 3.56

a The value is the mean of three determinations.

### 3.5 Forced degradation studies of SMV

Hydrolytic, oxidative and photo forced degradation were studied. In all cases, an appreciable SMV degradation was observed. Hydrolytic degradation was performed in the dark to cancel any photo degradation. The percentage of degradation of SMV was calculated according to the following equation:
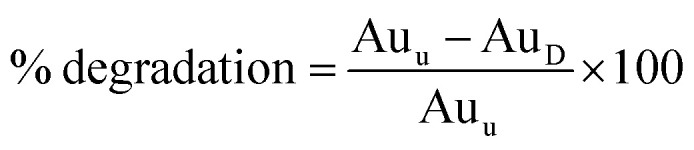
where; Au_u_ is the area under the peak of untreated stock drug solution and Au_D_ is the area under the peak of the drug in the degraded sample.

**Fig. 5:**
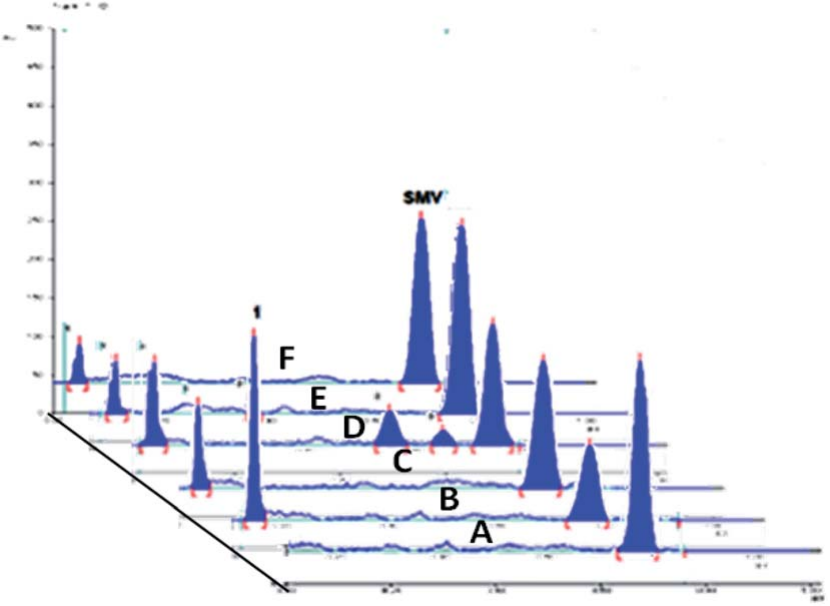
A 2D chromatogram of (A) 500 ng per spot SMV, (B) 1.0 M HCl acid-induced degradation, (C) 1.0 M NaOH base-induced degradation, (D) oxidative degradation with 30% v/v H_2_O_2_, (E) UV light degradation products, and (F) neutral hydrolysis degradation products.

**Fig. 6:**
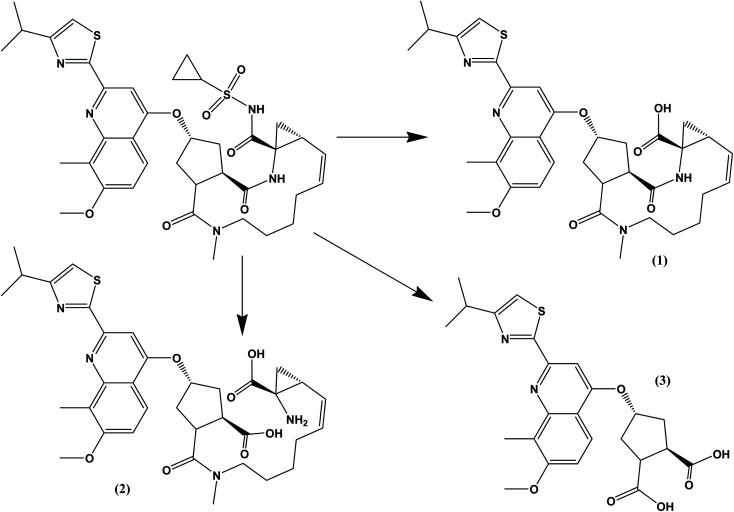
Possible pathways for the hydrolytic and oxidative degradations of SMV. Compound (1) was previously suggested as the oxidative product.

The drug contains several amide bonds and therefore, the drug is very highly liable for hydrolytic conditions. About 71.2% degradation of SMV was observed after its exposure to 1.0 M HCl at 60 °C for 2 h. In the case of alkaline degradation, the percentage of degradation was 39.8% using 1.0 M NaOH at 60 °C for 2 h. In both case, only one degradation product was observed at *R*_F_ = 0.03 which indicates the high polarity of the degradation product. Oxidative degradation by using 30% v/v H_2_O_2_ at 60 °C for 2 hours induced 51.3% degradation. A previously reported study^11^ claimed that only one oxidation product was formed. However, in the present study, three degradation products were observed on the chromatogram of the sample subjected to oxidative degradation. The same peak of the hydrolytic degradation was also observed in addition to two products appeared at *R*_F_ of 0.47 and 0.57. Although the extent of photochemical and neutral hydrolytic degradation are lower than the acidic hydrolytic, alkaline hydrolytic and oxidative routs, it still high. The percentages of degradation were 26.6 and 19.3% for photochemical degradation (UV irradiation at 254 nm for 48 h) and neutral hydrolysis (at 80 °C for 6 h), respectively. Again the peak at *R*_F_ = 0.03 was observed in both case with a minor peak at 0.29 for photochemical degradation. Typical densitograms obtained for SMV under different stress conditions are shown in (Fig. 5) and a summery for the results are presented in (Table 7). Based on this study, SMV should be protected from moisture, light and elevated temperature due to the high liability of the drug to all the studied stress degradation condition. Possible pathways for the degradation of SMV are presented in (Fig. 6).^10^

**Table 7:** Summary of degradation of SMV under different stress conditions

Degradation type	Condition	Number of degradation products (*R*_F_)	% degradation
Acidic	1.0 M HCl at 60 °C for 2 h	1 (0.03)	71.2%
Basic	1.0 M NaOH 60 °C for 2 h	1 (0.04)	39.8%
Oxidative	30% v/v H_2_O_2_ at 60 °C for 2 h	3 (0.03, 0.47, 0.57)	51.3%
UV light	UV irradiation at 254 nm for 48 h	2 (0.03, 0.29)	26.6%
Neutral	At 90 °C for 6 hours	1 (0.03)	19.3%

## 4. Conclusion

The developed HPTLC technique is specific, direct, fast and sensitive for determination of simeprevir in pharmaceutical formulations and spiked human plasma. The planned methodology exhibited smart advantage, such as the ability of direct sample application with none any derivatization, short analysis time, batch analysis and minimal solvent usage. Moreover, it does not involve the extensive cleanup or sample pretreatment which is routinely used in the liquid chromatographic methods. The proposed method clearly showed stability-indicating potential being able to satisfactorily quantify SMV simultaneously with its degradation products.

## Conflicts of interest

There are no conflicts to declare.

## Supplementary Material
